# Hydrogen-plasma-induced magnetocrystalline anisotropy ordering in self-assembled magnetic nanoparticle monolayers

**DOI:** 10.3762/bjnano.4.16

**Published:** 2013-03-04

**Authors:** Alexander Weddemann, Judith Meyer, Anna Regtmeier, Irina Janzen, Dieter Akemeier, Andreas Hütten

**Affiliations:** 1 Research Laboratory of Electronics, Massachusetts Institute of Technology, 77 Massachusetts Ave, Cambridge, MA 02139, USA; 2 Department of Physics, Thin Films and Physics of Nanostructures, Bielefeld University, PB 100131, 33501 Bielefeld, Germany

**Keywords:** dipolar particle coupling, magnetic nanoparticles, magnetocrystalline anisotropy, monolayers

## Abstract

Self-assembled two-dimensional arrays of either 14 nm hcp-Co or 6 nm ε-Co particle components were treated by hydrogen plasma for various exposure times. A change of hysteretic sample behavior depending on the treatment duration is reported, which can be divided in two time scales: oxygen reduction increases the particle magnetization during the first 20 min, which is followed by an alteration of the magnetic response shape. The latter depends on the respective particle species. Based on the Landau–Lifshitz equations for a discrete set of magnetic moments, we propose a model that relates the change of the hysteresis loops to a dipole-driven ordering of the magnetocrystalline easy axes within the particle plane due to the high spatial aspect ratio of the system.

## Introduction

Due to their wide range of applications in physical, biological and medical fields, magnetic nanoparticles have been thoroughly studied during the past few decades [1–2]. In this regard, various manufacturing techniques to synthesize particles with distinct magnetic properties [3–4] or specific biological surface coatings [5–6] have been established. Such nanocrystals have a nonzero magnetization at zero field because of finite-size effects. Nevertheless, due to their superparamagnetic nature, the effective magnetic moment of an ensemble of noninteracting magnetic nanoparticles is zero if there is no external field applied.

The situation changes if various types of interaction become important. A common example is given by ligand- or polymer-stabilized magnetic nanoparticles that tend to assemble in self-ordered two-dimensional arrays of high spatial symmetry [7–9] or various superstructures such as lines or rings [10–11]. In these systems, the magnetic coupling between individual particles increases the geometrical order of the assembly, which makes such patterns promising candidates for the design of novel data-storage devices [12]. A basic prerequisite for such an application is the high thermal stability of a magnetic state in order to maintain the magnetic configuration and not to lose the stored information.

In the case of a single particle, materials with a strong uniaxial magnetocrystalline anisotropy, such as face-centered tetragonal L1_0_ FePt alloyed particles, meet this requirement [12–13]. The magnetic-moment vector aligns with the easy axis due to energy minimization. In the transition from a single free particle to a closed monolayer, stray-field contributions of contiguous particles need to be taken into account. For such ensembles of interacting magnetic nanocrystals, not only the magnetocrystalline contribution, but also the magnetic coupling determines the stability of a given magnetic state. In particular, the most stable magnetic configuration is achieved whenever the magnetocrystalline axes of individual particle components are aligned parallel to the magnetization directions of the magnetic equilibrium state of the system itself.

If particles with low magnetocrystalline anisotropy are considered, the magnetic equilibrium state is mainly dominated by dipolar coupling. In this case, the magnetic-moment vectors do not tend to align with the easy axes. Instead, as long as the crystallographic orientation of the particles can rotate freely by some mechanism, the easy axes align with the magnetic-moment vectors in order to minimize the total energy. Thus, the stability of the initial equilibrium configuration is increased. We investigate the two-dimensional assemblies of Co nanoparticles of different crystallographic phases and sizes under the influence of a hydrogen plasma. We will show evidence for such an ordering of the magnetocrystalline easy axes and consequent stabilization of the corresponding magnetic equilibrium states. The experimental results will be compared to numerical calculations based on the idea that the plasma induces a process comparable to the time-dependent creep under tension in which the plasma acts as thermal activation.

## Experimental

Measurements were carried out with two different species of monodisperse Co particles, which will be referred to as sample I and sample II in the following. Sample I consists of particles with an average diameter *d*_I_ = 13.80 nm and a standard deviation of σ_I_ = 2.60 nm, while nanoparticles in sample II have a size of *d*_II_ = 6.09 nm at a standard deviation of σ_II_ = 1.14 nm. According to Hütten et al. [3], the smaller species are superparamagnetic while the larger contain a certain degree of ferromagnetic components.

### Sample preparation

Both samples were prepared in a procedure introduced by Puntes et al. [14] under airless conditions. For the synthesis of sample I, 65 µL (0.2 mmol) oleylamine was dissolved in 4 mL 1,2-dichlorobenzene. The solution was subsequently heated under reflux. Separately, 150 mg (0.44 mmol) dicobaltoctacarbonyl Co_2_(CO)_8_ was dissolved in 2 mL of 1,2-dichlorobenzene.

During vigorous stirring, the second solution was rapidly injected into the refluxing bath. After a reaction time of 1 h, the mixture was cooled to room temperature. For the synthesis of particle species II, 265 µL of a mixture of equal parts of oleylamine and oleic acid were dissolved in 4 mL 1,2-dichlorobenzene and heated to 180 °C. Similar to sample I, 180 mg (0.53 mmol) dicobaltoctacarbonyl Co_2_(CO)_8_ was dissolved in 2 mL of 1,2-dichlorobenzene and rapidly injected into the refluxing bath. After a reaction time of 15 min, the mixture was cooled to room temperature. Due to the different surfactants present during the particle formation, particles in sample I are stabilized by oleylamine, while species II present a composition of oleylamine and oleic acid on the surface.

**Figure 1:**
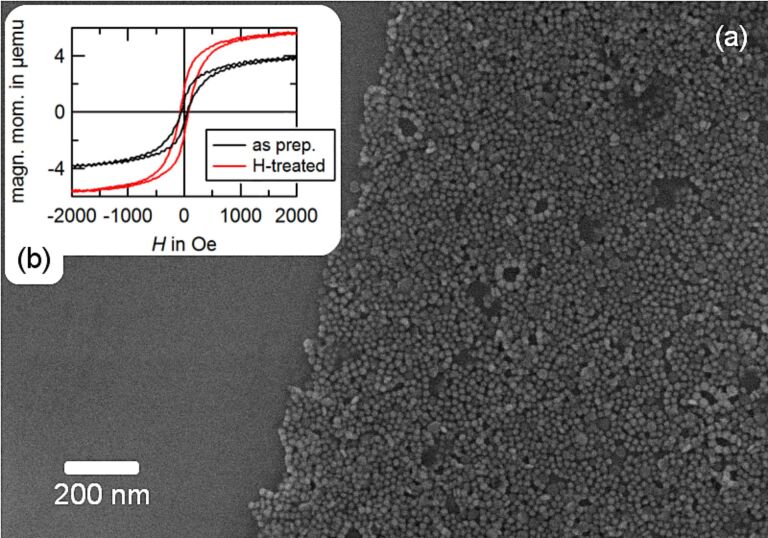
Monolayer of magnetic Co nanoparticles, species I. (a) SEM image of a two-dimensional particle assembly of 13.8 nm oleylamine-stabilized Co particles. (b) AGM measurements before and after hydrogen treatment.

In order to realize closed nanoparticle monolayers, a silicon wafer with a SiO_2_ layer of 500 nm thickness was dipped into the particle solution at an angle of 45°. The angle is experimentally chosen to provide the optimal deposition of closed particle monolayers onto the substrate. Upon evaporation of the liquid, particles remain immobile on the substrate surface. An example of a scanning electron microscopy image (SEM) taken from the resulting assemblies is shown in Figure 1a.

### Hydrogen plasma treatment

The self-assembled two-dimensional particle arrays were exposed to a soft hydrogen plasma (100 W) at room temperature under a pressure of 1.7 × 10^−3^ mbar for different exposure times. In order to analyze the influence of the plasma on the magnetic properties of the nanoparticles, alternating gradient magnetometer (AGM) measurements were performed before and after plasma treatment. Since a significant degree of oxidation can occur on very short time scales [15–16], the samples were covered in situ with a thin protective layer. These layers were deposited employing magnetron sputtering. For sample I and II, Ir and Pt were chosen as the respective layer materials; the layer thickness was set to 10 nm in both cases. Different materials were employed to ease the evaluation of the X-ray diffraction (XRD) measurements (see below).

**Figure 2:**
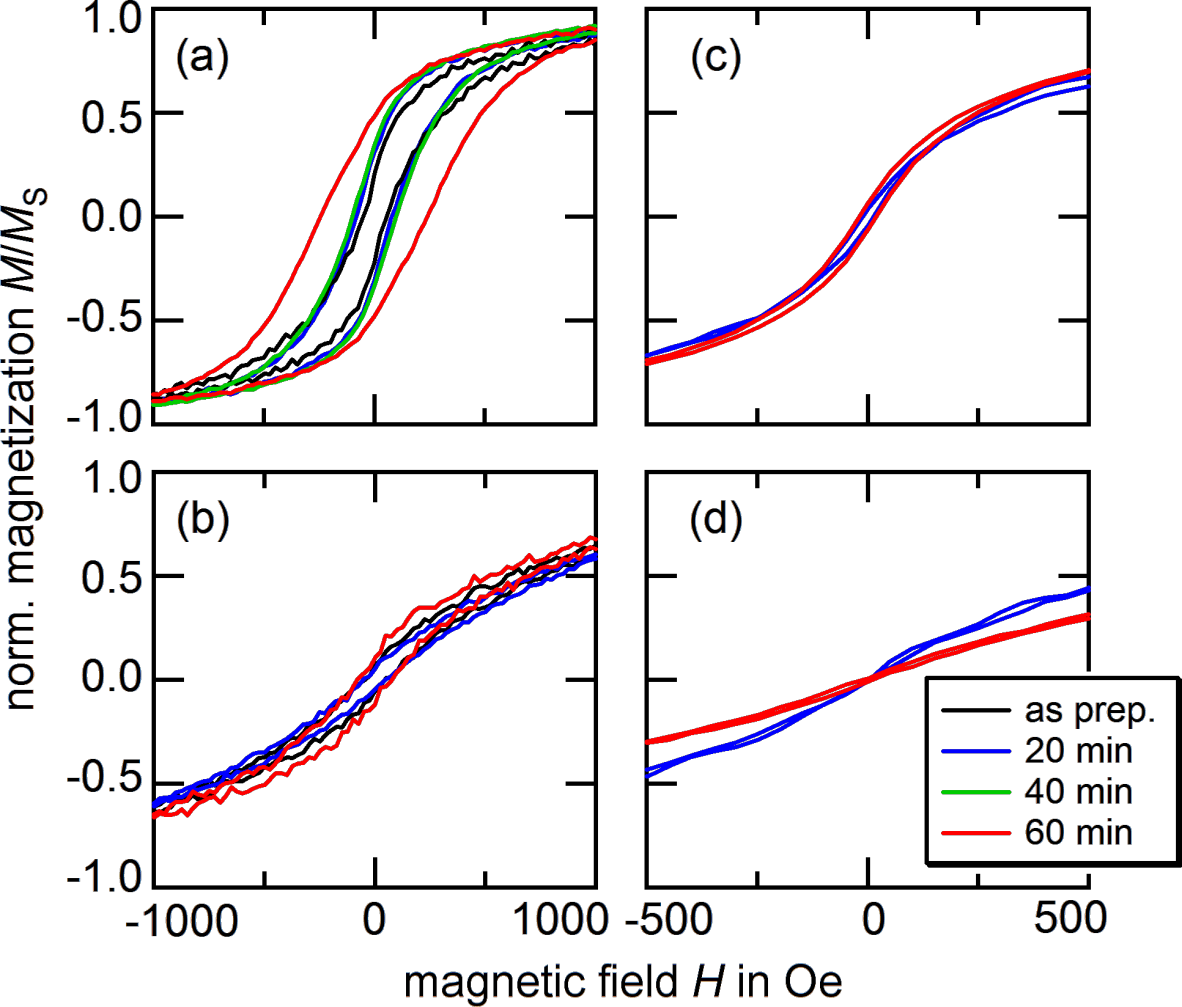
AGM measurements of nanoparticles assembled in monolayers after different exposure times to a hydrogen plasma. The results were obtained at room temperature. (a) and (b) show the behavior of particle species I for in-plane and out-of-plane external fields; (c) and (d) are the respective results for species II.

On short time scales of approximately 20 min, an oxygen reduction of the particle material is expected, which entails an increase of the saturation magnetization of the sample [17–19]. For a sample prepared with species I, an example of such an increase is shown in Figure 1b. After a time period longer than 20 min, no further increase of the magnetic moment can be observed. However, as shown in Figure 2, the shape of the measured hysteresis loops is altered with respect to the exposure time. With *M*_S_ being the saturation magnetization of the sample material, the normalized magnetization *M*/*M*_S_ is shown with respect to the applied magnetic field. Subplots (a) and (b) represent the behavior of sample I for in-plane and out-of-plane measurements, respectively, and (c) and (d) the corresponding results for species II. All measurements were carried out at room temperature.

As a measure of the magnetic properties, we evaluate the remanent magnetization *M*_R_, the coercive field *H*_C_, and the change of the normalized magnetization *M*/*M*_S_ at the magnetic field strength *H* = *H*_C_

[1]
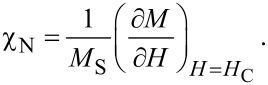


The evaluation of these parameters for in- and out-of-plane measurements are given in Table 1, with the respective indices || and 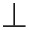.

**Table 1:** Characteristic magnetic data obtained from AGM measurements for sample I and II for different plasma treatment times. Measurements were taken at room temperature.

	*M*_R,||_ [*M*_S_]	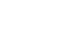 [M_S_]	*H*_C,||_ [Oe]	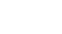 [Oe]	χ_N,||_ [mOe^−1^]	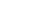 [mOe^−1^]

I, as prep.	0.200	0.059	56.72	44.40	2.95	1.43
I, 20 min	0.297	0.039	84.63	56.60	3.30	0.80
I, 40 min	0.333	0.067	100.51	63.17	3.38	0.82
I, 60 min	0.478	0.096	242.60	73.99	2.17	1.38
II, as prep.	0.028	0.010	10.69	10.02	2.65	1.01
II, 20 min	−0.018	0.007	−6.27	5.76	2.84	1.23
II, 40 min	0.004	0.021	1.27	20.64	3.01	1.02
II, 60 min	0.060	0.010	18.40	11.70	3.23	0.85

For in- and out-of-plane measurements of samples prepared with particle species I, we find increasing values for the remanent magnetization *M*_R_ and the coercive field *H*_C_ with longer treatment times. In contrast to these observations, the experiments carried out on species II show no clear tendency for these particular values. Instead, we find an increasing in-plane and a decreasing out-of-plane value for *χ*_N_, which cannot be reported for species I.

In order to ensure that the observed changes in the hysteretic behavior cannot be attributed to changes of the particle morphology due to the impact of the plasma, the particle shape was analyzed. The sample used for this analysis was prepared in a dropping procedure with species I and, consequently, exposed to the hydrogen plasma for three hours. The sample was covered by a thin Pt layer of 15 nm thickness to prevent oxidation of the Co particles. A scanning microscopy image taken along the particle plane reveals a situation similar to Figure 1. However, this observation does not exclude a deformation along the out-of-plane direction such as the flattening of the spheres towards ellipsoidal colloids.

**Figure 3:**
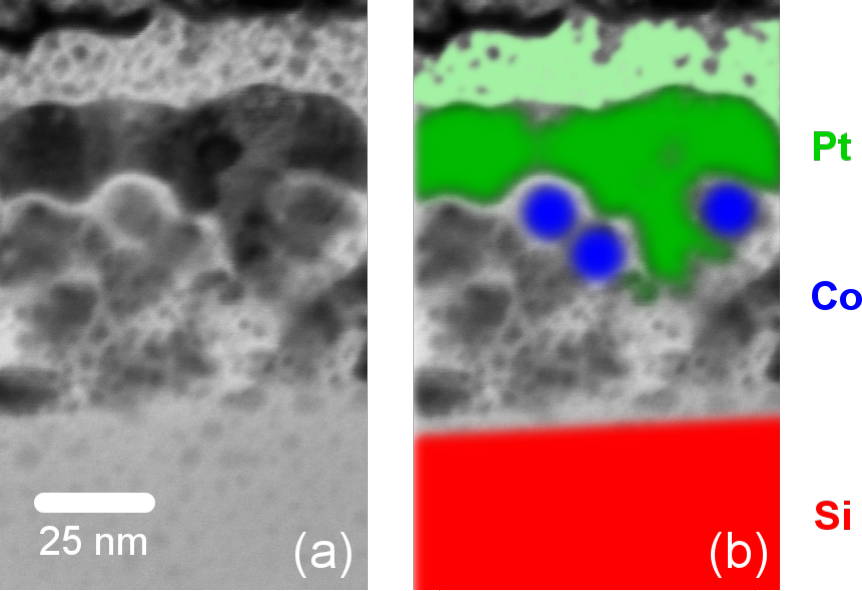
(a) STEM image of the cross section of a plasma-treated sample. (b) As highlighted, the Co particles maintain their spherical shape. The spots in the top Pt layer refer to carbon inclusions due to the preparation process.

For the imaging of a particle cross section along the out-of-plane axis, a thin sample lamella was prepared with a FEI Helios Dual Beam FIB by cutting through a suitable particle agglomeration. The lamella was subsequently thinned down to a thickness of 20 nm. In order to protect the particles from contamination and possible ablation by gallium ions, an additional thin protective layer of platinum was deposited with the electron beam before the preparation process. A scanning transition electron microscopy (STEM) image of the cross section is shown in Figure 3a, and the different material regimes are highlighted in Figure 3b to aid understanding. The area on the bottom of the figure (red) shows the Si wafer. Particles on top of the substrate (blue) are covered by a thin layer of Pt of approximately 15 nm thickness (green), to prevent oxidation, and an additional 15 nm Pt layer (bright green) deposited before cutting the lamella, to protect the sample from ablation by gallium ions. During the deposition, carbon inclusions are created, which can be seen as dark spots along the corresponding area. As highlighted in Figure 3b, Co particles maintain their spherical shape. Therefore, the observations described above cannot be related to topological changes.

### XRD measurements

By annealing wet-chemically synthesized FePt nanoparticles at a temperature of 600 °C, Antoniak et al. [20] found evidence for the partial formation of the chemically ordered L1_0_ state, which entailed a significant increase of the coercive field by a factor of 6 after thermal treatment. With the pure Co particles studied in this work, local composition variations within individual particles may not be at hand; however, Co particles can be found in the hcp-, fcc or ε-crystallographic phases [21]. In order to understand the different behaviors of sample I and II, the crystallographic structures were analyzed by XRD measurements before and after plasma treatment.

**Figure 4:**
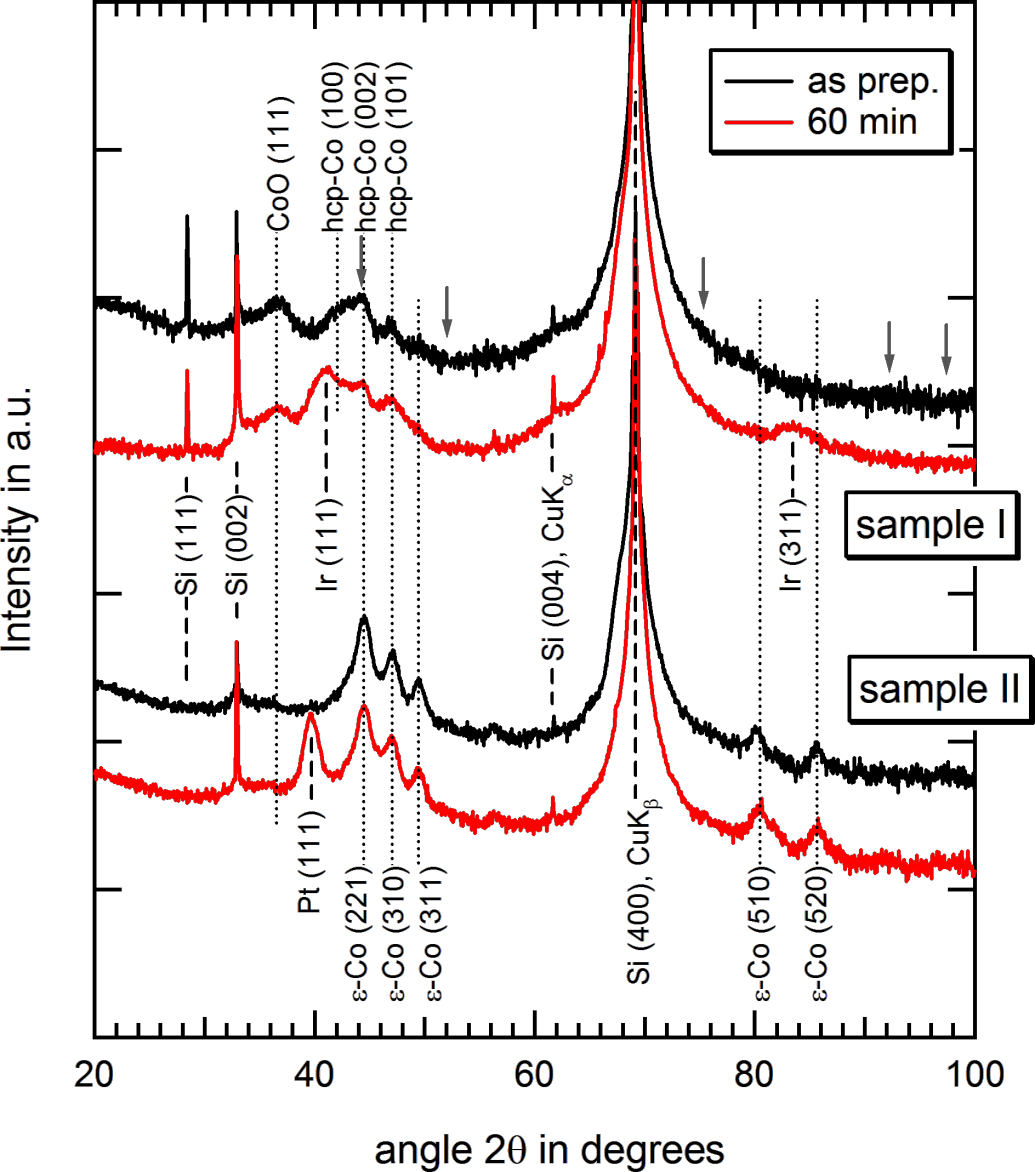
XRD measurements of sample I (top) and sample II (bottom) before (red) and after (blue) hydrogen-plasma treatment. Particle species I shows an hcp order while sample II is crystallized in the ε-phase. No evidence of an fcc-Co phase can be found; the gray arrows in the top plot indicate the expected peak positions. Further, each phase maintains its stability during plasma treatment.

The measurements reveal that species II can be found in an ε-phase, while sample I is ordered on an hcp-lattice (Figure 4). The arrows indicate the expected peak positions of the fcc-phase (44.22° (111), 51.52° (200), 75.86° (220), 92.23° (311), 97.66° (222)), which are not present in either sample. According to the XRD data, no phase transition during plasma exposure is observed. This is in accordance with the findings by Sun and Murray [22] who reported the transitions between the different crystallographic phases ε-Co → hcp-Co and hcp-Co → fcc-Co to have activation temperatures of about 300 and 500 °C, respectively.

### Numerical model

In order to obtain a qualitative understanding of the microscopic origin of the experimental findings, simulations of two-dimensional particle arrays are carried out. Since particles of the given size are superparamagnetic, they are homogeneously magnetized along their volume. Therefore, each individual particle may be approached by its magnetic moment 
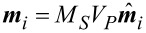
, with *M*_S_ being the saturation magnetization of the material, *V*_P_ the particle volume and 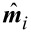 the angular components. The equilibrium state of a system of ferromagnetic components is a solution of [23]

[2]
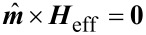


[3]
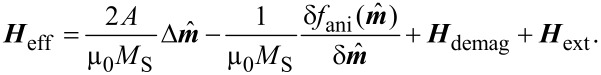


The first term of Equation 3 corresponds to the magnetic exchange energy with the exchange constant *A*. Since single-domain particles are considered, no variations of the magnetization can be found along the magnetic volume, and therefore, this contribution equals 0. The second term refers to magnetocrystalline anisotropy with *f*_ani_ being the anisotropy energy functional. In order to study the influence of anisotropy effects, we will assume two different types of anisotropy: (a) uniaxial anisotropy, in which the crystal structure has an energetically favorable direction, the easy axis ***k***; and (b) cubic anisotropy. The respective energy functionals are given by

[4]
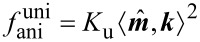


**Figure 5:**
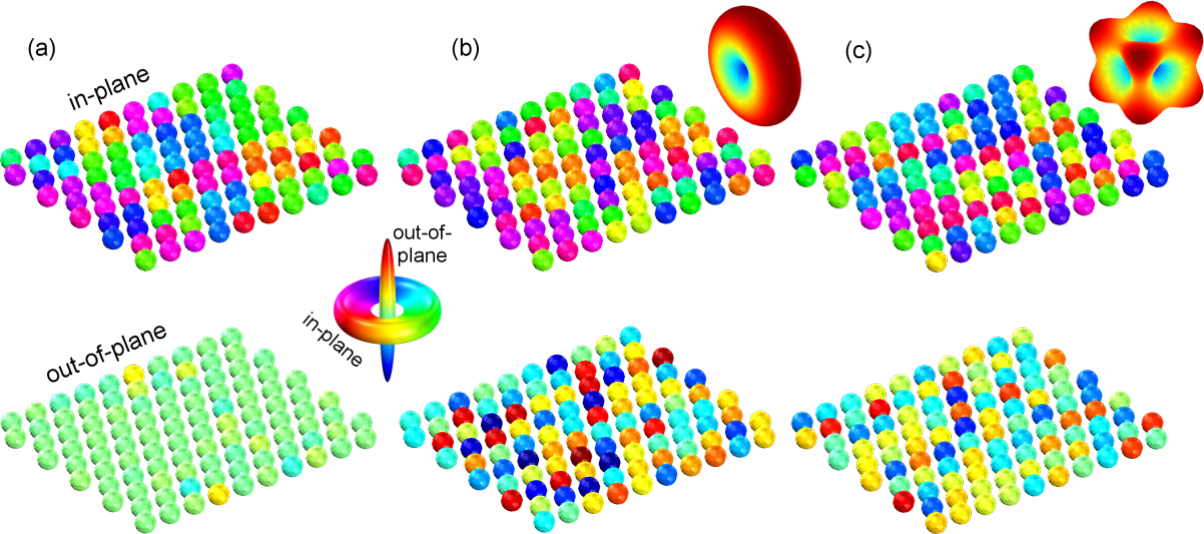
Equilibrium state of the model system. Particles of 13 nm size assembled in a hexagonal lattice with a grid constant of 18 nm and a saturation magnetization of *M*_S_ = 900 kA/m. Subplots show different anisotropy scenarios: (a) amorphous, (b) uniaxial and (c) cubic magnetocrystalline anisotropy. The surfaces in the upper-right corner of (b) and (c) represent the angular energy distribution of the respective anisotropy scenario where blue areas correspond to energy minima (easy axes) and red to maxima (hard axes). The upper plots present the in-plane component of the magnetic moments (color-code: disc), the lower ones the out-of-plane component (color-code: cone).

[5]
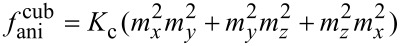


with the anisotropy constants *K*_u_, *K*_c_ and the Euclidean inner product 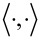. The corresponding energy surfaces in dependency on the solid angle are shown at the top of Figure 5 (see below).

A particle at position ***R****_i_* creates a magnetic stray field at every space point ***r***. Due to its magnetic single-domain structure, the magnetic field is described by a dipolar approximation. Therefore, the *j*-th particle at position ***R****_j_* feels a field given by the superposition of all other field contributions

[6]
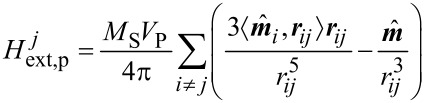


with ***r****_ij_* = ***R****_j_* − ***R****_i_* being the distance vector and *r_ij_* = |***r****_ij_*| its absolute value. Due to a rapid decrease with distance, not all particles need to be taken into account, but it is sufficient to restrict the analysis to contributions from particles at a distance smaller than 7.5 times the average particle radius of the system. This threshold value is in accordance with the findings of Schaller et al. [24]. The total external-field contribution acting on a particle is given by the sum of Equation 6 and an additional homogeneous-field contribution applied to bring particles to magnetic saturation. Finally, the third term of Equation 3 can be omitted since the demagnetization field ***H***_demag_ of a homogeneously magnetized sphere is antiparallel to the magnetization vector 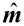 and, consequently, we always have 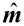 × ***H***_demag_ = 0.

### Assumptions

With 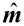 constant on each individual particle, Equation 3 is transformed from a set of partial differential equations to a set of ordinary ones. A solution is obtained by consideration of its time-dependent extension [25]

[7]
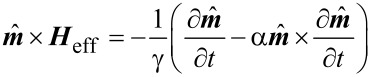


with γ the gyromagnetic ratio and α a dimensionless damping constant. The microscopic relaxation occurs on time scales significantly shorter than the time scales on which external fields change. Therefore, the microscopic dynamics are not in the scope of this work and the value of the damping parameter may be adjusted to provide a high numerical convergence rate. We chose α = 1 [26]. For the integration with respect to time, a backward differential formula of fifth order is applied. As a model system, we consider a two-dimensional, 10 × 10 particle lattice of hexagonal symmetry with a lattice constant of 18 nm, which was taken from the experiments. Furthermore, the particle diameter and magnetization are set to *d* = 13 nm and *M*_S_ = 900 kA/m [27], respectively. In order to analyze the influence of the magnetocrystalline anisotropy, uniaxial and cubic scenarios according to Equation 4 and Equation 5 with the respective choices of anisotropy constants *K*_u_ = 50, 100 and 150 kJ/m^3^ and *K*_c_ = 30 and 50 kJ/m^3^ are studied. The bulk values of fcc and hcp Co cubic anisotropy constants lie in the range of 27 to 45 kJ/m^3^ [28]. For particles at the edges of the lattice, periodic boundary conditions are employed.

Examples of the equilibrium states of such systems are shown in Figure 5, for different cases: (a) amorphous particles, (b) particles with a randomly oriented uniaxial anisotropy, *K*_u_ = 50 kJ/m^3^, and (c) particles with a randomly oriented cubic anisotropy, *K*_c_ = 30 kJ/m^3^. For each subplot, the upper part shows the in-plane magnetic component (color-code: disc) and the lower the out-of plane component (color-code: cone). The surfaces in the upper right corner of subplots (b) and (c) represent the angular energy distribution of uniaxial and cubic anisotropy, where blue areas correspond to the energy minima (easy axes) and red ones to maxima (hard axes).

For magnetically amorphous particles (Figure 5a), the spatial confinement in two dimensions entails the alignment of the magnetic moments parallel to the particle plane. Contiguous magnetic moments are likely to align parallel or antiparallel to each other. Such a configuration minimizes the stray-field energy of the system. For uniaxial anisotropy (Figure 5b), the magnetic moments partially follow the easy axis and, therefore, show a significantly higher *z*-component whenever the easy axis 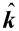 is perpendicular to the particle assembly. In comparison, with an increased number of such axes, the probability of an energetically favorable direction parallel to the particle plane is higher in the case of cubic magnetocrystalline anisotropy. Consequently, the *z*-component of individual magnetic moments lies between the amorphous and the uniaxial case (Figure 5c).

For the uniaxial settings, the distribution of the anisotropy vectors 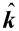 is chosen in three different ways:

1. equally random on the unit sphere in three dimensions,
2. equally random on the section of the three-dimensional unit sphere that includes an angle α with the *xy*-plane between −45° < α < 45°,
3. and equally random on the two-dimensional unit sphere in the *xy*-plane.

For the systems with cubic anisotropy, two different distributions are studied:

1. easy axes are randomly distributed,
2. one easy direction coincides with the axis perpendicular to the particle plane.

**Figure 6:**
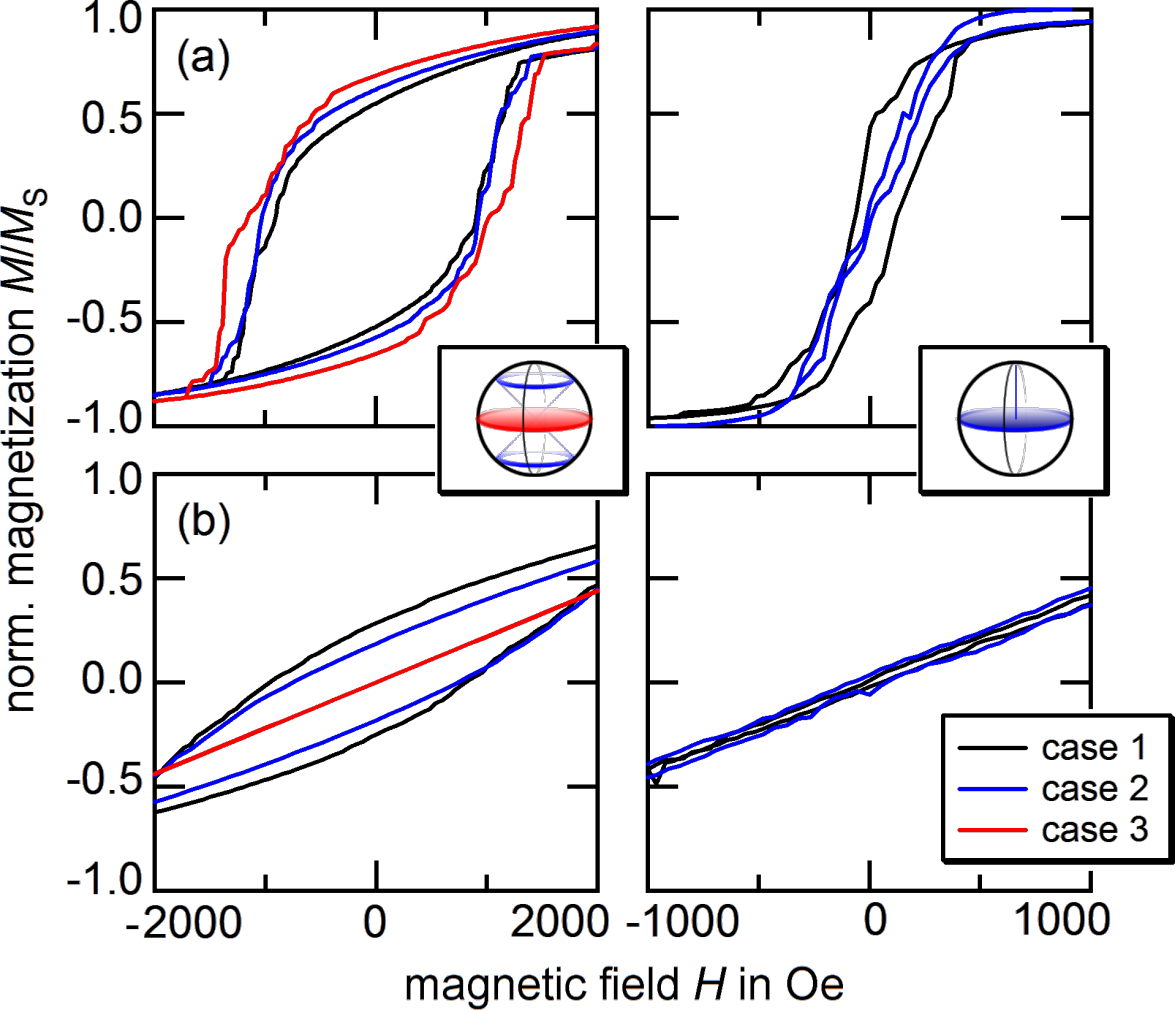
Hysteresis loops of the 10 × 10 hexagonal lattices shown in Figure 5 for different anisotropy cases obtained from numerical calculations. (a) and (b) show the behavior of particles with a uniaxial anisotropy and *K*_u_ = 10^5^ J/m^3^ for in-plane and out-of-plane external fields; (c) and (d) show the respective results for cubic anisotropy with *K*_c_ = 3 × 10^4^ J/m^3^. The insets visualize the different choices for the distribution of easy axes.

Both cases are schematically shown in the insets of Figure 6. These choices are motivated by the equilibrium state for amorphous particles aligning their magnetic moment parallel to the particle plane (Figure 5). After preparation of the samples, the magnetocrystalline orientations are randomly distributed along the sample. Similar to the microscopic ordering during annealing, see e.g. [29], the crystallographic orientation may change under plasma treatment following the magnetic stress induced by the stray fields of contiguous particles. The mechanism is comparable to mechanical creep under tension with the hydrogen plasma acting as the thermal actuator. With the stray-field energy minimum obtained for an in-plane magnetic configuration, the easy magnetocrystalline axes of individual particles should migrate into the particle plane, resembling the respective choices made above. In order to analyze the hysteretic behavior, an alternating external field ***H***_eff_ in the *x*- and *z*-direction is considered.

## Results and Discussion

Typical hysteresis loops, which result from the numerical analysis are shown in Figure 6; remanent magnetization *M*_R_, coercive field *H*_C_, and magnetization change at *H* = *H*_C_ are given in Table 2 for various magnetocrystalline anisotropy assumptions. We begin our discussion with the case of uniaxial anisotropy: For in-plane measurements, remanent magnetization and coercive field increase with decreasing average 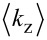. This is in agreement with the experimental findings. The increasing in-plane alignment of the magnetocrystalline easy axes results in a higher stability of the magnetic states and the particle assembly exhibits an increasingly harder magnetic behavior. For perpendicular external fields, the opposite tendency can be observed. The corresponding values drop down to zero and the system behaves similarly to a paramagnet. The effective anisotropy of geometrical and magnetocrystalline contributions has no longer an out-of-plane contribution, and therefore, the *z*-component of individual magnetic moments resembles a behavior similar to the amorphous state shown in Figure 5a, bottom. The derivative χ_N,||_ does not show a clear tendency for the in-plane evaluation, which is due to a step-like decrease/increase of the magnetization with respect to the applied field; the appearance of hard shoulders will be discussed below. For the out-of-plane case, a decreasing 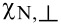 with decreasing 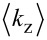 may be reported. These results are independent of the value of the anisotropy constant. However, with higher anisotropy constants, in-plane and out-of-plane data obtain similar values, i.e., the anisotropy energy becomes the dominant contribution and overcomes the dipolar coupling.

**Table 2:** Characteristic magnetic data obtained from numerical calculations for different anisotropy cases. The remnant magnetization of particle species II is close to 0, which indicates the superparamagnetic behavior as mentioned before. Sign inversions may be attributed to noise effects such as thermal contributions.

		*M*_R,||_ [*M*_S_]	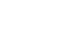 [*M*_S_]	*H*_C,||_ [Oe]	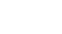 [Oe]	χ_N,||_ [mOe^−1^]	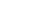 [mOe^−1^]

uniaxial 1	50	0.6485	0.1403	408.56	370.65	6.26	0.47
uniaxial 2	50	0.7084	0.1155	427.98	348.27	6.88	0.35
uniaxial 3	50	0.7095	≈0	473.42	≈0	3.41	0.34
uniaxial 1	100	0.5356	0.2673	906.03	884.72	2.97	0.34
uniaxial 2	100	0.5952	0.1841	976.00	746.60	3.67	0.30
uniaxial 3	100	0.6682	≈0	1087.49	≈0	1.04	0.22
uniaxial 1	150	0.5400	0.3366	1527.03	1534.88	3.48	0.40
uniaxial 2	150	0.6048	0.2429	1480.74	1210.44	2.60	0.23
uniaxial 3	150	0.6198	≈0	1661.77	≈0	1.36	0.17
cubic 1	30	0.4205	0.0151	92.95	36.35	4.67	0.49
cubic 2	30	0.4216	0.0573	225.26	169.82	4.84	0.47
cubic 1	50	0.0457	0.0467	9.93	80.34	9.93	0.46
cubic 2	50	0.0470	0.1266	20.13	404.84	2.26	0.45

For cubic systems, the components of remanent magnetization and coercive field are significantly lower. The higher number of easy axes provides a less restrictive energy landscape in the sense that distinct energy minima are separated by lower energy barriers, which entails a softer switching behavior. Depending on the choice of the anisotropy constant *K*_c_, the in-plane magnetization may increase or decrease with a higher order of the magnetocrystalline easy axes. For low values, switching between different magnetic configurations is enhanced, which coincides with the case of the ε-Co phase employed in species II [8].

In general, the obtained values for the remanent magnetization and coercive field are much higher than the experimental observations. Further, the hysteresis loops do not exhibit smooth characteristics but show multiple step-like jumps. These effects may be attributed to various simplifications of the simulations: (a) Temperature was not taken into account, which entails a higher stability (higher obtained values [30]). (b) In comparison to the experimental system, only a small number of particles was modeled. The appearance of small domains (compare Figure 5) that switch as a whole entails hard shoulders in the hysteresis curves. (c) The assumption of a perfect grid entails anisotropic response functions [31]. In the experimental realization, the data resemble the average taken over all measuring directions due to arbitrarily oriented particle grains. Finally, (d) a small degree of the sample area is not covered by a monolayer, but multilayers/particle clusters can be found instead. Even though they are only present on a very low area ratio, they may still contain a high number of particles. This particularity diminishes the observed effects and results in an increased 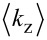 in these areas.

## Conclusion

The exposure of magnetic Co nanoparticles to hydrogen plasma entails an alteration of the magnetic sample response. From XRD analysis, we were able to conclude that the plasma has no impact on the crystallographic phase, and STEM images of the particle cross sections revealed an unaffected particle shape. By comparison to numerical data obtained by solving the stationary micromagnetic equations, we proposed a model for the influence of the plasma treatment on the microscopic structure. The magnetocrystalline easy axes of individual particles align with the stray field of contiguous nanocrystals. The process is comparable to the time-dependent creep under tension with the plasma acting as the thermal activation.

For uniaxial magnetocrystalline anisotropy, the migration of the magnetocrystalline easy axes results in an increase of the effective sample anisotropy, which entails a hard switching behavior for in-plane measurements and a soft paramagnetic one for out-of-plane measurements. For cubic symmetry, the in-plane hysteresis decreases over time due to a higher number of energy minima within the particle plane.
